# Contraceptive Counseling: Construction and Validation of Instrument—“5C Contraceptive Counseling”

**DOI:** 10.3390/healthcare12111088

**Published:** 2024-05-25

**Authors:** Sara Palma, Diogo Ayres-de-Campos, Mónica Antunes, Ricardo São-João, Maria Helena Presado

**Affiliations:** 1 Nursing School of Santarém, Polytechnic Institute of Santarém, 2005-075 Santarém, Portugal; 2 Nursing Research, Innovation and Development Center of Lisbon (CIDNUR), 1600-096 Lisbon, Portugalmhpresado@esel.pt (M.H.P.); 3 Faculty of Medicine, University of Lisbon, 1649-004 Lisbon, Portugal; 4 Garcia de Orta Hospital, ULS Almada/Seixal, 2805-267 Almada, Portugal; 5 Nursing Research Platform Lisbon of Health Research Center (CIIS), Portuguese Catholic University, 1649-023 Lisbon, Portugal; 6 School of Management and Technology, Polytechnic Institute of Santarém, 2001-904 Santarém, Portugal; 7 Statistics and Applications Center, University of Lisbon (CEAUL), 1749-016 Lisbon, Portugal; 8 Center for Global Studies, Universidade Aberta, 1250-100 Lisboa, Portugal; 9 Nursing School of Lisbon, 1600-190 Lisbon, Portugal

**Keywords:** contraception, contraceptive advice guide, counseling, decision making, shared, nurse–midwives, reproductive health

## Abstract

Introduction: Contraceptive illiteracy leads to non-adherence, discontinuation, and dissatisfaction with the method. Person-centered contraceptive counseling is based on quality care on a communicative basis that promotes shared decision-making, leading to a choice adapted to the woman’s needs, lifestyle, and health condition. We intend to build and validate an instrument that serves as a guide for quality contraceptive counseling, facilitating decision-making about contraceptive methods. Methods: We used the Delphi method in a total of two rounds. The content was validated through a panel of eighteen experts with experience in teaching, research in contraceptive counseling, and obstetric nursing. To assess the consensus and stability of the responses, two questionnaires were administered and the Content Validity Index and Content Validity Ratio were calculated. Results: The initial version of the guide, consisting of six indicators and thirty-five items, was submitted to the panel of experts to obtain consensus and stability from respondents (first round). The results showed a response rate of 66.0%; thirty-four indicators reached consensus and one did not reach consensus. Suggestions for modifying the indicator were received by the experts and incorporated in the next round. In the second round, the response rate increased to 78.0%. Two indicators were resubmitted, of which one was accepted. This resulted in the final version of the instrument, with six points and thirty-five items. Discussion: The guide proved to be a valid tool for nurse–midwives to provide quality contraceptive advice to women, allowing them to make autonomous and informed choices regarding their sexual and reproductive health.

## 1. Introduction

Sexual health encompasses a state of physical, emotional, mental, and social well-being, and is an integral part of the development of each individual’s personality. It not only encompasses certain aspects of reproductive health but also the possibility of having a satisfying and safe sexual life, being a fundamental human right closely linked to respect, protection of human rights, non-discrimination, privacy, confidentiality, and freedom from violence and coercion [1,2,3]. Included in these care services are access to health services, education, and information on family planning and contraception (modern, equitable, free, and tailored to the needs of its users), allowing for the conscious choice of a method that best suits the woman’s condition, enabling them to decide freely when and how many children they want to have, as well as the spacing between pregnancies. It is an essential requirement for reducing the number of unplanned pregnancies [1,4].

Contraceptives aim to prevent sexual intercourse from resulting in pregnancy, with the choice of method respecting the woman’s will, lifestyle, maternity plans, and health condition [5,6].

In Portugal, there are health policies that have shown improvements in sexual and reproductive health indicators, namely: (i) in the use of contraceptive methods, including methods that are less dependent, or not dependent on the user (ii) in the progressive reduction in the number of births in voluntary termination of pregnancy (TOP), and (iii) in reducing maternal complications due to unsafe abortions. The laws and policies in force protect the most vulnerable individuals, guaranteeing the right to information and quality, and accessible health services in an equitable manner for all citizens.

Although the free distribution of contraceptive methods and equal access to services are legislated, there is a lack of knowledge about contraceptives, which can lead to nonadherence, discontinuity, and dissatisfaction with the method, which is associated with difficulties in accessing family planning services [7,8], which can result in a number of unplanned and unwanted pregnancies and voluntary TOPs. The COVID-19 pandemic has accentuated some inequalities in accessibility to healthcare and contraceptives (namely long-acting reversible ones) [9,10], due to the need to redistribute healthcare professionals due to the high number of people infected with COVID-19, which conditioned the response of services, and reduced family planning consultations [9], an aspect that can be proven through the number of voluntary TOPs performed.

Since the decriminalization of the voluntary TOP law in 2007, a decreasing trend has been observed in the number of abortions performed, with a reversal of this trend between 2021 and 2022 where 15,870 voluntary TOPs were performed in Portugal, at the woman’s option, up to 10 weeks of gestation, representing an increase of 15.0% compared to the previous year [10]. It is known that 93.0% of women opt for contraceptive use after a voluntary TOP, and 98.0% of those who undergo repeat abortions were already using contraception [9,10,11]. The repetition of a TOP may be related to the high failure rate of user-dependent methods and the contraceptive counseling strategies employed post-abortion [10].

As a voluntary TOP is considered a public health problem with psychological, economic, and social implications [8], it is imperative to develop effective forms of contraceptive counseling.

Often, women’s contraceptive choices are based on cultural factors and method accessibility [12], with the majority (76.0%) showing a preference for oral hormonal contraception [10,11]. However, 84.0% of them acknowledge having forgotten to take the pill, and 47.0% intend to switch to a method that does not require daily intake [13]. From the evidence presented, it is clear that women intend to use a contraceptive, but their satisfaction and adherence remain unknown, prompting concern to investigate access to family planning appointments, the type of counseling provided by healthcare professionals, and the appropriate use of contraceptive methods by women [11].

In an observational and retrospective study involving 1287 women, a significant number were found to use either oral hormonal contraceptives or no method at all [9]. These results may be related to a lack of information about family planning and contraceptive methods, as well as difficulties in accessing healthcare services and the fragility of reproductive health education initiatives that address the needs and preferences of women, especially in socially vulnerable groups (such as young people, economically deprived classes, those with low educational attainment, and migrants) [9,14,15,16].

Women with lower levels of education, young age, and limited economic resources tend to have more difficulty internalizing and utilizing information provided by healthcare professionals, leading them to use contraceptives that are more accessible but not always the most suitable for their health condition, preferences, and lifestyle. This often results in feelings of dissatisfaction with the contraceptive method chosen and eventual discontinuation [15,17,18].

Objectives three and five of the Sustainable Development Goals [19] aim to reduce inequalities and promote health equity, including family planning, information, and education, as well as the integration of reproductive health into national strategies and programs in a prioritized, universal manner with political, social, economic, and environmental implications. Thus, to provide quality contraceptive counseling that promotes the use of a contraceptive method suitable for the specificity and individuality of each individual, it is necessary to build a trusting relationship between the healthcare professional and the person, allowing them to express their beliefs, attitudes, desires, and fears, based on effective communication, trust, privacy, and confidentiality, enabling autonomous, informed, and coercion-free choice [20].

A systematic literature review [15] addressing the impact of contraceptive counseling on method choice after an induced abortion revealed that women receiving contraceptive counseling are more likely to choose a contraceptive method post-abortion [21,22,23], exhibit a lower risk of unintended pregnancy [22,24], have increased adherence [25,26], and are associated with the quality of information shared by trained professionals during contraceptive counseling sessions [27]. Professionals with higher levels of training dedicate more time to counseling, influencing the quality of care provided, method choice, and adherence [28].

Associated with women’s difficulty in choosing a contraceptive is the low health literacy and inadequate search for reliable information [16,29], which is why counseling should be provided by trained professionals, focused on the individual needs and uniqueness of each woman [30].

Since being sexually active requires autonomy based on solid knowledge to make appropriate choices and avoid situations that endanger people’s health and lives [31], healthcare professionals and nurse–midwives, in particular, should promote women’s empowerment in adopting healthy lifestyles in the context of sexual health and family planning [32], increasing their health literacy for informed decision-making and choice of a method according to each person’s life goals and health conception, facilitating adherence, satisfaction, and method continuity.

Ensuring quality care involves efforts that result in effectiveness, equity, and accessibility to health services and professionals [33,34,35].

Demystifying preconceived ideas about contraceptive methods, understanding life plans in the context of sexual and reproductive health, assessing personal and family obstetric and gynecological history, finding ways to prevent sexually transmitted infections, and evaluating satisfaction and adherence to the chosen method are crucial [11,36].

In order for healthcare professionals to provide quality contraceptive counseling, they must follow a set of steps that should not be overlooked in promoting shared decision-making and adherence to contraceptives. In this sense, the present research originated from the construction and validation of a contraceptive counseling instrument aimed at guiding healthcare professionals when conducting contraceptive counseling. The aim was to construct a guiding instrument of good care practices that would assist nurse–midwives in their activities when promoting sexual and reproductive health and in empowering women for shared decision-making regarding the choice of a contraceptive method.

## 2. Materials and Methods

In this methodological study conducted between April and June 2021, we planned, constructed, and validated a guiding script for contraceptive counseling. We utilized the Delphi method, as it allows for the gathering of a consensus on a specific subject or theme, stimulating discussion among experts [37]. This study integrates a mixed exploratory research approach and follows the principles of the Helsinki Declaration. It was submitted and approved by the Ethics Committee (No. 52/2020) on 24 July 2020. All participants were informed about the nature and purpose of the study, providing informed consent through questionnaire responses.

This study comprises several stages, from selecting experts, constructing and sending the initial questionnaire, receiving responses, analysis, and constructing and sending a second questionnaire with feedback from the first [38]. This process is ongoing until the desired levels of stability and consensus in responses are achieved. To assess the consensus and stability of responses, we applied the Content Validity Index (CVI) calculation and the Content Validity Ratio (CVR) calculation [39,40]. The analysis and statistical procedures were carried out using the IBM SPSS Statistics version 28^®^ program.

In the initial phase, we developed a questionnaire with proposed interventions to be implemented by nurse–midwives during contraceptive counseling sessions. We employed a mnemonic as a facilitation strategy to stimulate memory and assist nurse–midwives in recalling the phases to be followed in contraceptive counseling sessions. Since it is a script to be applied in a contraception session, we used the letter “C”, distributed across five phases that we deemed essential in quality counseling.

The “5Cs” of Contraceptive Counseling:

1. Building a Relationship of Trust;
2. Understanding Knowledge and Beliefs;
3. Empowering for Effective Contraception;
4. Implementing Contraceptive Measures/Choices;
5. Continuing Surveillance and Monitoring.

This set of ideas came from the results obtained through a systematic review of the literature on the impact of contraceptive counseling after voluntary TOP [14], which was constantly updated in the search for new evidence on the topic of contraceptive counseling. These ideas also came from the categories that emerged from the analysis of testimonies from a focus group carried out with nurse–midwives on how they carried out contraceptive counseling sessions, and the aspects that promoted women’s contraceptive decision-making after an abortion [16]. This group supports the need to have an instrument that guides them on how to provide contraceptive counseling in the sessions. After its construction and before being applied to the panel of experts, its understanding was validated by a nurse–midwife and a professor specializing in the area.

The questionnaire was divided into two parts: one for characterizing the experts (age, sex, academic qualification, and professional experience in the field), and the second part consisting of 6 questions with 35 items.

To answer each of the questions requested, we used a 5-point Likert Scale that assessed the degree of agreement with the contents to be covered in the questionnaire, where 1 corresponded to “Totally Disagree” and 5 to “Totally Agree”. We consider 3 as the “Neither Agree Nor Disagree” option. For each question, a synthesis was made containing the main ideas to be considered. At the end of each question, space was reserved for providing suggestions that could lead to new items in the questionnaire for the next round [37]. For this panel of experts, we consider that we have reached consensus and stability when we obtain a minimum agreement rate of 80% [41] for each of the indicators evaluated and an RVC greater than or equal to 0.4 [42]. To calculate the CVI and CVR, we considered the responses of the experts who mentioned having “Totally Agree” and “Agree” to the questions asked.

For this panel of experts, we consider that we have reached consensus and stability when we obtain a minimum agreement rate of 80% (CVI) [41] for each of the indicators evaluated and a CVR greater than or equal to 0.4 [42]. To calculate the CVR and CVI, we considered the responses of the experts who mentioned having “Totally Agree” and “Agree” to the questions asked.

The criteria used to define an expert in the field of interest were: (a) nurse–midwives and nursing lecturers in maternal and obstetric health; (b) professional experience in clinical practice, teaching, research, and/or scientific knowledge in contraceptive counseling; (c) interest in the topic under study; and (d) participation in working groups on contraception and publications in the area of the topic under study.

The selection of nurse–midwives was based on a close relationship with the population to whom they provide care, having legislated skills that allow them to care for women within the family and community within the scope of family planning and in situations of abortion and interventions developed in the provision of preventive care in the area of family planning and contraceptive counseling [43]. In the Portuguese reality shows, nurse–midwives and community midwives are often primarily responsible for contraceptive advice. For the reasons presented, we included nurse–midwives in the panel of experts, although we recognize that the guide can be used by all professionals who provide contraceptive advice, regardless of the context.

In total, 35 experts were identified and invited, by convenience, and contacted via email to participate in the study. Consent was given by responding to the questionnaire, which was conducted through the Google Forms platform. Each expert received a link via email to respond to the questionnaire.

The number of experts invited to participate was evaluated to obtain a robust sample and ensure the quality of responses was not compromised. The literature suggests an abstention rate between 30.0 and 50.0% in the first round and from 20.0 to 30.0% in the second round [44]. Anonymity and confidentiality of the responses provided by the participants were ensured.

## 3. Results

Consensus and stability of responses were achieved after two rounds. The first round took place between 28 April and 18 May 2021. In the first round of the Delphi panel, out of the 35 experts invited to participate, 23 responded (66.0%), which can be considered a significant number generating relevant information [37].

The second round occurred between 30 May and 4 June 2021, with the new questionnaire sent to the 23 participants who responded to the first round, obtaining 18 responses (78.0%). In both rounds, the CVI and the CVR were calculated to understand the level of consensus of the responses given.

The 23 responding experts had the following characteristics (Table 1):

### 3.1. First Delphi Round

Regarding the response to the first question “Phases of Contraceptive Counseling Consultation”, the expert group was asked about their opinion regarding the organization of the consultation according to the mnemonic of the “5Cs” of Contraceptive Counseling. In response to this question, a concordance rate of 96.0% was observed. The following five questions focused on validating each of the “C” of the guide.

In the first C—“Building Trusting Relationships”—the nurse’s interventions consisted of seven items. Of the seven items, we identified a CVI of over 80.0% for all items and a CVR of over 0.40.

The second C—“Understanding Knowledge and Beliefs”—with twelve items, showed a concordance rate of 100.0% for all items, a CVI of 100.0%, and a CVR of 1.

The third C—“Empowering for Effective Contraception”—presented nine items. As with the previous “C”, the responses from the experts ranged from “Fully Agree” to “Agree”, with a concordance rate between 96.0 and 100.0%, a CVI of over 80% for all items, and a CVR of over 0.40.

The fourth C—“Implementing Contraceptive Measures/Choices”—consists of five items, which maintain the same trend in response distribution as the previous ones, with 98.0% of the responses falling between “Fully Agree” and “Agree”. It presented a concordance rate between 96.0 and 100.0%, representing a CVI of over 80.0% for all items and a CVR of over 0.40.

The fifth and final “C”—“Continuing Surveillance and Monitoring”—was divided into seven items. Of the seven items, six showed a concordance rate above 80.0%, except for the item “Assessing Satisfaction and Adherence to the Selected Method Annually”, where a concordance rate of 78.0% was obtained, with a CVI of 78.0%. Although the CVR is 0.56, consensus was not reached on this indicator. Thus, this item was reformulated according to the suggestions presented by the experts and re-sent for further evaluation in a new round.

### 3.2. Second Delphi Round

The second questionnaire followed the same principles as the first one. Two new items were presented with a brief description and evaluated using a Likert scale ranging from 1 to 5 points, where the lowest value corresponded to “Completely Disagree” and the highest to “Completely Agree”. The second round took place between 30 May and 4 June 2021. The new questionnaire was sent to the same participants who responded to the first survey, through the Google Forms platform. In the second round, 18 (78.0%) out of 23 experts responded. As with the first questionnaire, we calculated the CVI and CVR to understand the level of consensus of the given responses. Regarding the question “Assessing Satisfaction and Adherence to the Selected Method Annually—Preferably by Teleconsultation”, we obtained a concordance rate of 61.0%, with a CVI of 0.61 and a CVR of 0.22; consensus was not reached on this indicator, so we considered removing it. In the question “Assessing Satisfaction and Adherence to the Selected Method Annually—Face-to-face, in situations of associated pathology (hypertension, epilepsy...) or after a situation identified in teleconsultation (unsatisfaction with the selected method...)”, the CVI calculation was 94.0% and the CVR was 0.88, so the item was included.

After tabulating the new data compared to the previously obtained ones, and the respective statistical analysis, we found a concordance of 97.0%, with CVI values between 91.0 and 100.0% and a CVR between 0.65 and 1, and the Delphi rounds were closed.

In the final version of the instrument with guidelines for contraceptive counseling, all topics of items 1C, 2C, 3C, and 4C were retained as they showed consensus above 80.0% in all items with an RVC always above 0.4. In 5C, one of the items was removed and replaced by another which allowed for a high level of consensus among the experts. The final version of the instrument is presented below (Table 2).

## 4. Discussion

Several steps were undertaken through this study: the selection of experts, construction and sending of the first questionnaire, receipt of responses, analysis, and the construction and sending of a second questionnaire with feedback from the first [38]. This process continued until the desired levels of stability and consensus in responses were achieved through the calculation of CVI and CVR [39,40].

The guide is in line with scientific evidence and the needs expressed by the obstetric nurses who perform contraceptive counseling [15,16,33].

During contraceptive counseling, the woman’s prior knowledge of contraception, the method she intends to use, and the reasons for this choice should be assessed. Guidelines should be provided covering the functioning, use, side effects, risks, benefits, and return to fertility after discontinuation of the method. Studies recommend the use of instruments with supplementary information to incorporate the messages conveyed and assist in the decision-making process, during and after the consultation (e.g., brochures, catalogs, mobile applications, etc.) [20,29,45].

This guiding instrument for best practices in contraceptive counseling serves as a guide for healthcare professionals in promoting sexual and reproductive health and empowering women to make shared decisions regarding contraceptive methods. For healthcare professionals to conduct quality contraceptive counseling, they must follow a set of steps that should not be overlooked in promoting shared decision-making and adherence to contraception, namely: (1) Building Trusting Relationships; (2) Understanding Knowledge and Beliefs; (3) Empowering for Effective Contraception; (4) Implementing Measures/Contraceptive Choices; and (5) Continuing Surveillance and Monitoring.

It aligns with studies recommending the use of supplementary information tools to incorporate transmitted messages and assist in the decision-making process during and after consultation [20,29,45].

Studies demonstrate that women who receive contraceptive counseling choose a contraceptive method according to their needs, preferences, and lifestyle [21,22,23], present a lower risk of unwanted pregnancy [22,23], and increase adherence and satisfaction with the selected method [25,26].

Associated with women’s difficulty in choosing contraception is low literacy [16,29], so the quality of information shared by trained professionals [27] influences the quality of care provided and increases literacy, choice, and adherence to the method [28], which is why counseling should be centered on the needs and individuality of women [30].

The Delphi method has limitations such as the lack of guidance and standards on quality, complexity, how to interpret/analyze results, and how to select experts. As the procedure depends on the quality of feedback provided by experts, careful analysis of responses is a major responsibility of the researcher. For experts’ responses to be independent, it is necessary to ensure that experts do not come into contact with each other. Another limiting aspect is the slowness and difficulty in carrying out the method, associated with the difficulty that participants have in maintaining a long-term commitment, which may be one of the reasons for the decrease in participants between rounds [37]. The fact that we addressed these limitations and difficulties allowed a clear vision of how to overcome these aspects, a crucial part of guaranteeing valid and reliable results.

## 5. Conclusions

The systematic literature review and analysis of interviews with a focus group of nurse–midwives conducting contraceptive counseling conducted prior to the development of the guide highlighted that the process of shared decision-making, centered on the individual during contraceptive counseling, promotes literacy, autonomy, choice, adherence, and satisfaction with a method, as it is tailored to the needs, preferences, and lifestyle of the woman. However, this requires comprehensive counseling conducted by trained professionals.

The development of this instrument contributes to a practice of quality care based on scientific evidence and promotes sexual and reproductive health by enabling individuals to choose a contraceptive method that suits their health conditions, plans, and lifestyles.

We strongly believe that this instrument is valid for all health professionals who, in their activity, provide contraceptive advice, whether in a community, hospital, or school context.

## Figures and Tables

**Table 1:** Characterization of expert panel participants.

N° of Experts	Average Age	Years of Professional Experience in the Field	Sex	Academic Qualifications	Professional Activity
23	49 years	12.5 years	Female	Master: 47.8% Nurse–midwife: 34.8% Doctoral: 17.4%	Nurse–midwives: 78.0% University lecturers: 22.0%

**Table 2:** Guidelines for contraceptive counseling.

The 5Cs of Contraceptive Counseling	
1C Building Trust Relationship	Create an Atmosphere of Closeness Be a Good Listener Consider Non-Verbal Aspects of Communication Encourage the Individual to Express Their Feelings, Doubts, and Fears Without Judgment Understand the Maternity Plan Use Language Suitable to the Individual Organize the Physical Space of the Location to Avoid Communication Barriers
2C Understanding Knowledge and Beliefs	Understand Previous Contraceptive Experiences Identify Previously Used Contraceptives Assess Previous Contraceptive Method Adherence Evaluate Contraceptive Knowledge Identify Preferences Regarding Contraception Assess Suitability of Contraception to Lifestyle Inquire About Concerns Regarding Contraceptive Use Evaluate Beliefs and Myths Regarding Contraceptive Use Assess Motivation for Contraceptive Use Encourage Discussion on the Benefits of Contraceptive Use Inquire About Perceived Risks of Non-Contraceptive Use Understand Factors That May Affect Continuation of Selected Method
3C Empowerment for Effective Contraception	Clarify Myths and Beliefs Meet Their Expectations and Needs Inform About All Contraceptive Options Available in Portugal—Use Support Tools and Digital Platforms Explain the Mode of Action, Effectiveness, Duration, How to Use, and Possible Side Effects of Contraceptives Assess the Risks/Benefits of Contraceptive Use Validate What Has Been Learned Encourage Informed Decision-Making by the Woman Provide Supportive Information Materials—Brochures and Websites Allow Time for Decision-Making
4C Implement Contraceptive Measures/Choices	Assist in Selecting the Appropriate Method Ask the Woman to Identify Barriers or Obstacles That May Compromise the Decision to Discontinue the Method Instruct on Monitoring Signs and Symptoms That May Lead to Method Discontinuation Demonstrate Availability Through Support Networks for Additional Clarifications—Telephone or Email Ensure the Woman’s Accountability in Informed Method Selection
5th C Continuing Surveillance and Monitoring	Schedule Follow-up After Method Selection Through Scheduling of In-Person Consultation Schedule Follow-up After Method Selection Through Scheduling of Teleconsultation Evaluate Satisfaction and Adherence to Selected Method After 3 Months Evaluate Satisfaction and Adherence to Selected Method After 6 Months Evaluate Satisfaction and Adherence to Selected Method Annually—In-Person, in situations of associated pathology (hypertension, epilepsy...) or after identification of dissatisfaction with the selected method in teleconsultation Provide Option to Change Contraceptive Method, According to Need Promote Screenings (Cervical Cancer, Breast Cancer, and Sexually Transmitted Infections)

## Data Availability

Data are contained within the article.
